# Data-Efficient Training of Gaussian Process Regression Models for Indoor Visible Light Positioning

**DOI:** 10.3390/s24248027

**Published:** 2024-12-16

**Authors:** Jie Wu, Rui Xu, Runhui Huang, Xuezhi Hong

**Affiliations:** South China Academy of Advanced Optoelectronics, South China Normal University, Guangzhou 510006, China; 2022024038@m.scnu.edu.cn (J.W.); 2021024024@m.scnu.edu.cn (R.X.); 2023024119@m.scnu.edu.cn (R.H.)

**Keywords:** visible light positioning (VLP), Gaussian process regression (GPR), active learning (AL), supervised learning (SL), training

## Abstract

A data-efficient training method, namely Q-AL-GPR, is proposed for visible light positioning (VLP) systems with Gaussian process regression (GPR). The proposed method employs the methodology of active learning (AL) to progressively update the effective training dataset with data of low similarity to the existing one. A detailed explanation of the principle of the proposed methods is given. The experimental study is carried out in a three-dimensional GPR-VLP system. The results show the superiority of the proposed method over both the conventional training method based on random draw and a previously proposed line-based AL training method. The impacts of the parameter of active learning on the performance of the GPR-VLP are also presented via experimental investigation, which shows that (1) the proposed training method outperforms the conventional one regardless of the number of final effective training data (E), especially for a small/moderate effective training dataset, (2) a moderate step size (*k*) should be chosen for updating the effective training dataset to balance the positioning accuracy and computational complexity, and (3) due to the interplay of the reliability of the initialized GPR model and the flexibility in reshaping such a model via active learning, the number of initial effective training data (*m*) should be optimized. In terms of data efficiency in training, the required number of training data can be reduced by ~27.8% by Q-AL-GPR for a mean positioning accuracy of 3 cm when compared with GPR. The CDF analysis shows that with the proposed training method, the 97th percentile positioning error of GPR-VLP with 300 training data is reduced from 11.8 cm to 7.5 cm, which corresponds to a ~36.4% improvement in positioning accuracy.

## 1. Introduction

Accurate knowledge of the user’s location is a prerequisite for high-quality location-based services (LBSs) which enable a variety of important functions in the era of the Internet of Things, such as asset monitoring and automatic scheduling, product adaptation, service recommendation, etc. To achieve accurate localization in the indoor environment where the satellite-based positioning method cannot work properly due to signal blockage, visible light positioning (VLP) based on the visible light communication technology which can leverage the ubiquitous indoor lighting infrastructure has been proposed and intensively studied [1]. Compared with the radio-frequency-based indoor positioning technologies, VLP based on the optical carrier with a much shorter wavelength is more robust against multi-path fading and electromagnetic interference. Previous work has shown a centimeter-level accuracy can be achieved by VLP [1], which outperforms the conventional radio-frequency-based counterpart (e.g., Wi-Fi and Bluetooth) by a large margin [2,3].

To achieve high positioning accuracy with VLP, a carefully designed positioning algorithm is indispensable. Recent studies show that VLP algorithms based on supervised machine learning (SL) (e.g., artificial neural network (ANN) [4], polynomial regression (PR) [5], k-nearest neighbors (KNN) [6], decision tree (DT), random forest (RF) [7], support vector machine (SVM) [8], and Gaussian process regression (GPR) [9,10,11,12,13]), which have a powerful nonlinear data processing capability, offer superior performance over the conventional positioning algorithms based on trilateration/triangulation using the Lambertian radiation model [14]. For example, the study in [10] shows that, in a received signal strength (RSS)-based VLP system, the GPR positioning algorithm is significantly better than that of the conventional propagation-model-based approach which suffers from model mismatch in practical applications. Similar findings have also been reported in [11] which shows that the data-driven GPR algorithm offers more robust and accurate positioning results than the conventional analytical Angle-of-Arrival multilateration-based algorithm. In SL-VLP, a customized model is created via training based on a labeled dataset (i.e., training data collected at known positions) and used to estimate the position of users at an unknown location via inference. For some SL algorithms (e.g., ANN and PR), the training and inference processes are decoupled as they are conducted at offline and online stages, respectively. In such a case, the computational complexity of position estimation remains unchanged even if a larger dataset is used in training to enhance the accuracy of the SL model. However, for many other SL algorithms, mostly non-parametric algorithms (e.g., RF, SVM, KNN, and GPR), the training and inference processes are coupled, which means that the complexities of both processes increase for a larger training dataset. This leads to a dilemma in these coupled SL-VLP methods where a large training dataset is favored for creating an accurate data-driven model, yet it increases the computational burden for the position estimation at every unknown location. It should be noted that efforts have been made to reduce the complexity of the training of SL-VLP systems [5,15,16,17]. Nevertheless, these works cannot solve the above dilemma because either only the decoupled SL-VLP method is considered [5,15] or the number of training data in the coupled SL-VLP method is not reduced [16,17].

Since different coupled SL methods may be developed based on quite different principles/mechanisms, the solution to avoid such a dilemma in general should be devised specifically for a certain SL positioning algorithm. In this paper, we target the GPR-based VLP system and propose a data-efficient training method to reduce the number of training data without compromising the accuracy of the GPR model in positioning. The reason for choosing GPR is that it is a probabilistic model that can simultaneously estimate the position and report the corresponding confidence of estimation [18], which is not possible for the other SL method based on a deterministic model. The unique probabilistic nature of the GPR model also makes it a valid tool for data augmentation for SL (e.g., NN in [15] and fingerprinting in [12])-based indoor positioning algorithms. Previous studies have shown that GPR outperforms the conventional propagation-model-based method [10,11] and some other SL methods (e.g., multi-layer perceptrons [13]) in terms of positioning accuracy. However, the computational resources and time required by GPR increase significantly when a large training dataset is employed, which negatively affects the performance of positioning accuracy [12]. In other words, the data efficiency in the training of the GPR model needs to be improved to solve the conflict between the positioning accuracy and the computational complexity. In this regard, we propose a data-efficient training dataset construction method for GPR-based VLP systems. The proposed method incorporates the idea of active learning [16,19,20] to progressively refine the data in a collected training dataset according to the data similarity reflected in the “confidence” output from the GPR model. A quadrant-based active learning method is devised to further enhance the data-efficiency in training which in turn reduces the complexity in positioning performed at every location. The experiment of a three-dimensional VLP system is carried out to evaluate the performance of the proposed method. Optimization of the parameter of active learning is also realized experimentally. The results show that the number of training data can be effectively reduced by the new training method which outperforms both the conventional training method and the line-based training method which also employs active learning under the same number of training data.

The contribution of our work is summarized as follows:

(a) A novel training dataset construction method based on active learning is proposed to reduce the size of the training dataset without compromising the positioning accuracy for VLP with GPR. The higher data efficiency in training offered by the proposed method can reduce the computational complexity of the positioning stage with GPR;
(b) The effectiveness of the proposed method in improving the data efficiency in training is proved experimentally in a 3D VLP system for both scenarios with and without the receiver tilt;
(c) The impacts of parameters of active learning on the performance of the proposed method have been studied via comprehensive experimental investigations, which gives insights into the optimization of the proposed method.

## 2. Principle of Data-Efficient Training in GPR-VLP

In GPR-VLP, the GPR model takes in the feature of received signals (*s*) and outputs the estimation of position (*p*) corresponding to the measurement. Without loss of generality, in this study, the received signal strength (RSS) of four carriers of different frequencies (i.e., $s=\left(s_{1},s_{2},s_{3},s_{4}\right)$) and the three-dimensional coordinates (i.e., $p=(l_{X},l_{Y},l_{Z}$) are used as the model’s input and output, respectively. To generate an accurate GPR model, a set of data is used for training which is denoted as follows:

(1) $$D=\left\{\left(s_{i},p_{i}\right)|i=1,\ldots ,N\right\}$$

where $s_{i}$ and ${\;p}_{i}$ are the RSS and label (i.e., position) of the *i*th training sample, respectively. As a non-parametric model, the training process of a GPR model is not conducted explicitly as an independent procedure before the inference process. Instead, the training and inference are coupled according to a multi-variate Gaussian process. Specially, the unknown location $p_{T}$ can be expressed as a Gaussian process conditioned on the known vector $P_{D}$ as follows:

(2) $$p\left(p_{T}|P_{D}\right)\sim N\left(\mu_{*},\sigma_{*}^{2}\right)$$

The mean $\mu_{*}$ and variance $\sigma_{*}^{2}$ of this distribution are as follows:

(3) $$\mu_{*}={\hat{p}}_{T}=K\left(s_{T},S_{D}\right){K\left(S_{D},S_{D}\right)}^{-1}P_{D}$$

(4) $$\sigma_{*}^{2}=K\left(s_{T},s_{T}\right)-K\left(s_{T},S_{D}\right){K\left(S_{D},S_{D}\right)}^{-1}K\left(S_{D},s_{T}\right)$$

where $s_{T}$ is the RSS measured at the unknown location $p_{T}$, and $S_{D}=\left(s_{1},s_{2},\ldots {,s}_{N}\right)$ and $P_{D}=\left(p_{1},p_{2},\ldots {,p}_{N}\right)$ are the RSS and position vectors of *N* training data, respectively. The $\mu_{*}$ and $\sigma_{*}^{2}$ can be interpreted as the estimated position ${\hat{p}}_{T}$ and the confidence of such estimation by the GPR model. The K(X, Y) in (3) and (4) is a matrix/vector with a dimension of *length*(X) (i.e., length of vector X) by *length*(Y) (i.e., length of vector Y). The element on the *i*th row and *j*th column of K(X, Y) depends only on the *i*th element of X $\left(i.e.,\;x_{i}\right)$ and *j*th element of Y $\left(i.e.,\;y_{i}\right)$ according to a certain kernel function $k\left(x_{i},y_{j}\right)$. The isotropic radial basis function $k\left(x_{i},y_{j}\right)=\exp\left(-\frac{{\left|x_{i}-y_{j}\right|}^{2}}{{2l}^{2}}\right)$ with a positive hyper-parameter *l* is used as the kernel function here as we find it offers good performance in a simple form.

It is obvious that the position estimation at every unknown location requires the evaluation of (3) with a measurement $s_{T}$ to obtain ${\hat{p}}_{T}$. The computational complexity of matrix inversion to obtain ${K\left(S_{D},S_{D}\right)}^{-1}$ is $O\left(N^{3}\right)$. Even if ${K\left(S_{D},S_{D}\right)}^{-1}$ is calculated offline, the computational complexity of (3) still scales proportionally with *N*. For the estimation confidence in (4), the computational complexity scales proportionally with *N*^2^ after excluding the matrix inversion. Therefore, the computational burden at every test location increases if a larger training dataset is employed in GPR to enhance the positioning accuracy. To reduce the complexity of positioning without compromising the positioning accuracy, a data-efficient training method is needed.

Algorithm 1 shows the pseudo-code of our proposed data-efficient training method based on active learning for GPR-VLP systems, which is denoted as “AL-GPR” in the rest of this paper. The first step is to divide the training dataset D into two sets: an initial effective training dataset $D_{e}$ with *m* randomly drawn data and the complement dataset $D_{*}.$ Then, the “similarity” among each s in $D_{*}$ and all data contained in $D_{e}$ is evaluated based on (4) using $D_{e}$ as the effective training dataset. After that, *k* data in $D_{*}$ which correspond to the *k* lowest confidence outputs are added to the effective training dataset (i.e., updating $D_{e}$) since these data have the lowest level of similarity to those included in the current GPR model. The effective training dataset $D_{e}$ is updated iteratively following the above process until the amount of data in $D_{e}$ reaches a preset value E. Finally, we use $D_{e}$ as the training dataset and perform positioning with measurement $s_{T}$ at any test location using (3) and (4).

For the initialization step in active learning, it is preferred that the data in the initialized $D_{e}$ have low similarity to each other to enhance the generality of the initialized GPR model. However, the random drawing method in Algorithm 1 does not naturally guarantee that, especially when D is not sampled uniformly in the whole test space or the initial size is small. To further improve the data efficiency in the training of the GPR model, a quadrant-based active learning method is proposed by modifying the initialization step in Algorithm 1 (* Note that the AL process is decoupled from the positioning stage. It only needs to be conducted once before the positioning stage. The operator |·| denotes the number of samples in a dataset). The test space is divided into four quadrants of equal size. A total number of $\left\lfloor m/4\right\rfloor$ data is randomly drawn from one quadrant according to their labels, where $\left\lfloor x\right\rfloor$ stands for the nearest integer value less than or equal to *x*. In this way, the data in the initialized $D_{e}$ are evenly distributed in the test space from a quadrant point of view. The pseudo-code of the modified algorithm (namely, Q-AL-GPR) is summarized in Algorithm 2 (* Note that the AL process is decoupled from the positioning stage. It only needs to be conducted once before the positioning stage.).
**Algorithm 1** GPR-VLP with Data-Efficient Training Based on Active Learning (AL-GPR)**Input:** training dataset D, the numbers of the initial and final effective training data (m and E, respectively), kernel function k$,\;\mathrm{measurement}\;s_{T}$ at an unknown location. **Output:**$\;\mathrm{estimated}\;\mathrm{position}\;{\hat{p}}_{T}$ and its confidence $\sigma_{T}^{2}.$ * Active Learning:  **Initialization:**
Divide D$\;\mathrm{into}\;\mathrm{an}\;\mathrm{initial}\;\mathrm{effective}\;\mathrm{training}\;\mathrm{dataset}\;D_{e}^{0}$$(\left|D_{e}^{0}\right|=m$) with m data randomly drawn from D$\;\mathrm{and}\;\mathrm{the}\;\mathrm{corresponding}\;\mathrm{complement}\;\mathrm{dataset}\;D_{*}^{0}\;(\left|D_{e}^{0}\right|=\left|D\right|-m).$ **While**$\;\mathrm{the}\;\mathrm{number}\;\mathrm{of}\;\mathrm{data}\;\mathrm{in}\;D_{e}^{i}$ after ith iteration is no larger than E (i.e., $\left|D_{e}^{i}\right|<$ E) **do** 1. evaluate the similarity of data in $D_{*}^{i}$ to $D_{e}^{i}$$\;\mathrm{using}\;(4)\;\mathrm{with}\;D_{e}^{i}$$\;\mathrm{as}\;\mathrm{the}\;\mathrm{training}\;\mathrm{dataset}.\;\mathrm{To}\;\mathrm{be}\;\mathrm{specific},\;\mathrm{for}\;\mathrm{each}\;\mathrm{data}\;d_{x}=\left(s_{x},p_{x}\right)\in D_{*}^{i}$$,\;\mathrm{calculate}\;\sigma_{x}^{2}=K\left(s_{x},s_{x}\right)-K\left(s_{x},S_{D_{e}^{i}}\right){K\left(S_{D_{e}^{i}},S_{D_{e}^{i}}\right)}^{-1}K\left(S_{D_{e}^{i}},s_{x}\right)$. $2.\;\mathrm{update}\;D_{e}^{i}$$\;\mathrm{and}\;D_{*}^{i}$ $\mathrm{by}\;\mathrm{moving}\;k\;\mathrm{data}\;\mathrm{with}\;k\;\mathrm{lowest}\;\mathrm{similarity}\;\mathrm{levels}\;\mathrm{from}\;D_{*}^{i}$ $\mathrm{to}\;D_{e}^{i}$$.\;\mathrm{Mathematically},\;\mathrm{the}\;\mathrm{step}\;\mathrm{is}\;\mathrm{conducted}\;\mathrm{as}\;\mathrm{follows}:\;D_{e}^{\left(i+1\right)}=D_{e}^{i}\cup \{d_{x}\in D_{*}^{i}\;with\;{k\;lowest\;\sigma }_{x}^{2}\;\}$$,\;D_{*}^{\left(i+1\right)}=\{d_{x}\in D_{*}^{i}\;with\;(\left|D_{*}^{i}\right|-{k)\;highest\;\sigma }_{x}^{2}\;\}$$.\;\mathrm{The}\;\mathrm{size}\;\mathrm{of}\;\mathrm{the}\;\mathrm{two}\;\mathrm{datasets}\;\mathrm{after}\;\mathrm{the}\;\mathrm{update}\;\mathrm{are}\;\left|D_{e}^{\left(i+1\right)}\right|=\left|D_{e}^{i}\right|+k\;and\;\left|D_{*}^{\left(i+1\right)}\right|=\left|D_{*}^{i}\right|-k$. 3. *i* = *i* + 1. **End**  **AL Output:**
$\mathrm{The}\;\mathrm{final}\;\mathrm{effective}\;\mathrm{training}\;\mathrm{dataset}\;D_{e}$$\;\mathrm{from}\;\mathrm{the}\;\mathrm{final}\;\mathrm{iteration}.\;\mathrm{Note}\;\left|D_{e}\right|=$ E. **Positioning Stage:** Calculate the estimated position ${\hat{p}}_{T}$ and the confidence of estimation $\sigma_{T}^{2}$$\;\mathrm{for}\;\mathrm{the}\;\mathrm{measured}\;\mathrm{RSS}\;s_{T}$ at an unknown location using GPR based on (3) and (4) with $D_{e}$ as the training dataset.

**Algorithm 2** GPR-VLP under Data-Efficient Training with Quadrant-Based Active Learning (Q-AL-GPR)  Input/output and all steps are the same as Algorithm 1 except for the initialization step in Active Learning. * Active Learning:  **Initialization:**
Divide D$\;\mathrm{into}\;\mathrm{an}\;\mathrm{initial}\;\mathrm{effective}\;\mathrm{training}\;\mathrm{dataset}\;D_{e}^{0}(\left|D_{e}^{0}\right|=m)$$\;\mathrm{and}\;\mathrm{the}\;\mathrm{corresponding}\;\mathrm{complement}\;\mathrm{dataset}\;D_{*}^{0}\;\left(\left|D_{e}^{0}\right|=\left|D\right|-m\right).$$\;\mathrm{For}\;\mathrm{each}\;\mathrm{of}\;\mathrm{the}\;\mathrm{four}\;\mathrm{quadrants}\;\mathrm{of}\;\mathrm{the}\;\mathrm{test}\;\mathrm{space},\;\left\lfloor m/4\right\rfloor$$\;\mathrm{data}\;\mathrm{are}\;\mathrm{randomly}\;\mathrm{drawn}\;\mathrm{according}\;\mathrm{to}\;\mathrm{their}\;\mathrm{labels}\;\mathrm{to}\;\mathrm{build}\;D_{e}^{0}$.  **Other steps:** The same as Algorithm 1. **Positioning Stage:** The same as Algorithm 1.

The AL-GPR and Q-AL-GPR use AL to construct a training dataset with data of low similarities to improve the data efficiency. The AL introduces additional complexity when compared with the random sampling method used in the conventional GPR. However, the construction of the training dataset only requires a one-time effort, which means that the AL only needs to be executed once for all positioning tasks in the future rather than on a per-task basis. Therefore, the overhead induced by AL becomes negligible from a long-term perspective as more positioning tasks share it. On the other hand, the complexity of the positioning stage scales proportionally with the size of the training dataset for both the conventional GPR and AL-GPR/Q-AL-GPR as they use the same procedures for positioning. As we will show in the next section, a smaller number of training data can be used by AL-GPR/Q-AL-GPR to achieve the same level of positioning accuracy. The computational complexity of the positioning stage of AL-GPR/Q-AL-GPR is lower than that of the conventional GPR. Unlike the construction of a training dataset which is realized only once for all positioning tasks, the positioning stage needs to be executed on a per-task basis. The advantage of AL-GPR/Q-AL-GPR in terms of the computational complexity of the positioning stage becomes even more appealing from a long-term perspective due to the accumulation of savings in complexity when more tasks are performed.

## 3. Experiments and Results

**Figure 1:**
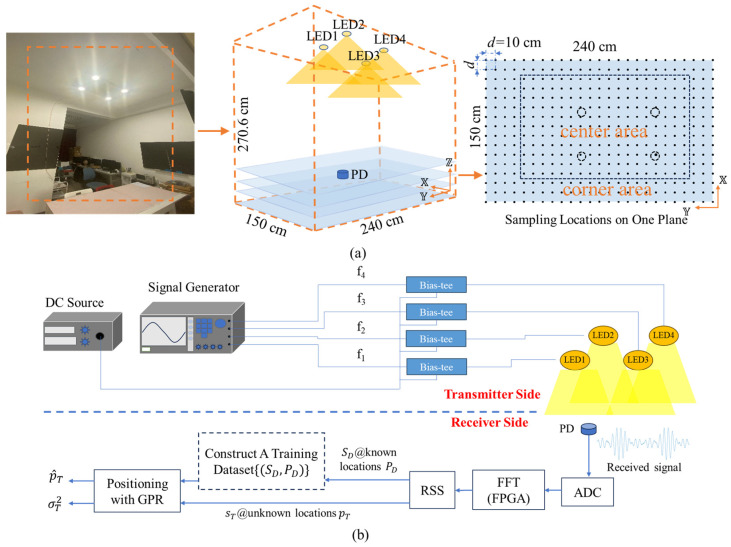
(**a**): A picture of the three-dimensional VLP testbed. A total number of 1600 locations evenly distributed on four planes of different heights are used for data collection in the test. The dotted circles and solid dots on the rightmost figure show the projection of four LEDs and sampling locations on one of the four planes, respectively. The inner and outer area divided by the dashed line corresponds to the “center” and “corner” cases, respectively. (**b**): Schematic diagrams of the 3D VLP system. Note that the training dataset only needs to be constructed once for all positioning tasks at unknown locations in the future.

To investigate the performance of the proposed data-efficient training methods in GPR-VLP systems, a three-dimensional VLP experiment is carried out. The number of LEDs is chosen to be four, which is larger than the minimum number required for the 3D VLP (i.e., three), to provide appropriate coverage of lighting and positioning service simultaneously. More LEDs with an appropriate design of distribution can be employed to enhance the system’s robustness against shadowing. The performance of the conventional GPR without AL in training is also evaluated for comparison. In the case of conventional GPR, the training dataset is constructed by sampling at random locations according to the uniform distribution in the positioning space. For conciseness, the conventional GPR is denoted as “GPR” in the rest of this paper. We would like to emphasize that GPR, AL-GPR, and Q-AL-GPR only differ in the way of constructing the training dataset (see Algorithms 1 and 2) but use the same estimation procedures according to (3) and (4). Figure 1a shows a picture of the VLP testbed with dimensions of 150 cm × 240 cm × 270.6 cm. Four light-emitting diodes (LEDs) which serve as both the light source for illumination and the beacon for VLP are installed on the ceiling with coordinates [48, 61, 270.6], [48, 137.5, 270.6], [95.5, 61, 270.6], and [95.5, 137.5, 270.6], respectively. Note that the asymmetrical arrangement of LEDs is caused by the layout of the room where a door/corridor is on the left side. The impact of different arrangements of LEDs on the performance of VLP based on GPR is not the focus of this work and is left for future study. To illustrate the physical layer of the VLP system, Figure 1b shows the schematic diagrams of the transmitter and receiver. At the transmitter side, each LED is driven by the signal from a Bias-tee which combines the sinusoidal signal with a unique frequency (400/500/600/700 kHz) from an electrical signal generator and the signal from a direct-current source. The modulated optical signal from the four LEDs is received by a photodiode (PD) at the receiver. The electrical signal from the PD is a result of the overlapping of four sinusoid signals of different magnitudes and phase delays (see the insert of Figure 1b). A photodiode (PD) together with an analog-to-digital converter (ADC) is used after the PD to sample and digitalize the received signal. The RSS is measured at the aforementioned frequencies after the fast Fourier transform of the time domain signal using a field programmable gate array (FPGA). RSS data are sampled at 1600 different locations which are evenly distributed on four planes (i.e., on a grid with a spacing *d* of 10 cm) at different heights (0/23/43/63 cm). Additionally, 70% of the collected data (i.e., *N* = 1120) are used as D. The remaining 30% (i.e., 480) are used as test data to evaluate the positioning error. The positioning error ε is defined as follows:

(5) $$\varepsilon =\left|p-\hat{p}\right|=\sqrt{{\left(l_{X}-{\hat{l}}_{X}\right)}^{2}+{\left(l_{Y}-{\hat{l}}_{Y}\right)}^{2}+{\left(l_{Z}-{\hat{l}}_{Z}\right)}^{2}}$$

where $p=\left(l_{X},l_{Y},l_{Z}\right)$ and $\hat{p}=\left({\hat{l}}_{X},{\hat{l}}_{Y},{\hat{l}}_{Z}\right)$ are the true and estimated coordinates of the test location, respectively.

**Figure 2:**
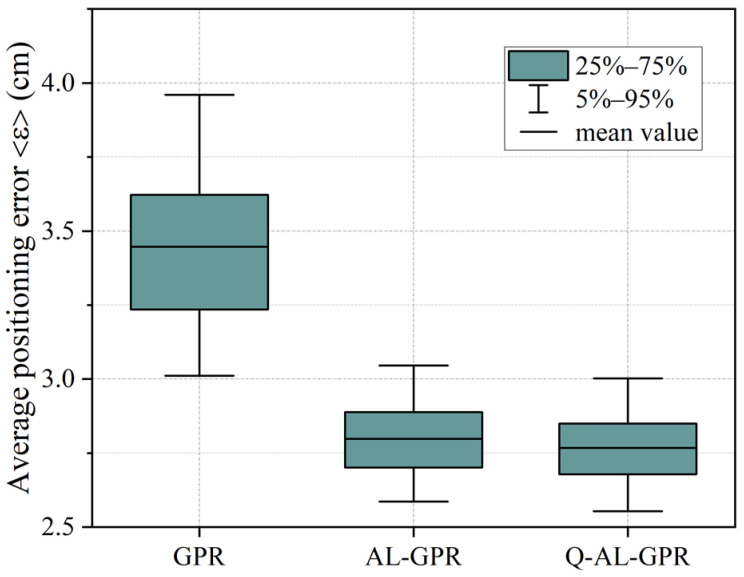
Statistics of the average positioning error $\langle \varepsilon \rangle$ of 480 random test locations under different training methods after 1000 runs.

Figure 2 shows the statistics of the average positioning error $\langle \varepsilon \rangle$ under different training methods after 1000 runs. The mean value together with the 5–95% and 25–75% intervals of $\langle \varepsilon \rangle$ are shown for the three training methods. In each run, the collected data are randomly split into training and test datasets with a fixed ratio of 7:3. The average position error $\langle \varepsilon \rangle$ of 480 random test locations is calculated for each run. For both AL-GPR and Q-AL-GPR, the initial number (*m*) and final number (E) of effective training data are set to 160 and 300, respectively. The step size (*k*) for the update of the effective training dataset is set to 28, which corresponds to five iterations in AL. Note that the conventional GPR uses all data in D for training. The mean values of the empirical distributions for the three training methods are shown by three vertical dashed lines, respectively. Compared with the GPR, the average positioning error is significantly reduced by the two data-efficient training methods. When AL-GPR (Q-AL-GPR) is employed instead of GPR, the mean value of $\langle \varepsilon \rangle$ can be reduced from 3.46 cm to 2.8 cm (2.76 cm). The widths of the 5–95% and 25–75% intervals shown in Figure 2 imply that $\langle \varepsilon \rangle$ is less dispersed when active learning is introduced. This is consistent with the measured variances of $\langle \varepsilon \rangle$ which are 8.77 mm^2^, 2.04 mm^2^, and 1.83 mm^2^ for GPR, AL-GPR, and Q-AL-GPR, respectively.

Table 1 shows the mean and variance of the empirical distribution for the two data-efficient training methods under different numbers of initial data (*m*). It is obvious that the Q-AL-GPR slightly outperforms the AL-GPR regardless of the value of *m*, which shows the effectiveness of the improved initialization method. The advantage of Q-AL-GPR over AL-GPR is more obvious for a smaller *m* since the accuracy of the initialized model is more sensitive to the choice of training data when its size is small.

As shown by the results of AL-GPR and Q-AL-GPR, which only differ in the initialization step, the choice of the initial effective training dataset indeed affects the performance of the GPR model. To give a more comprehensive analysis of such impact, we have conducted further studies to show the performance of GPR with AL under more choices of initial effective training data. Specifically, besides the two existing cases (i.e., random drawn for AL-GPR in Algorithm 1 and quadrant-based uniform drawn for Q-AL-GPR in Algorithm 2), two additional cases that use the data in the central locations (denoted as “central”) and the corner locations (denoted as “corner”) are selected as the initial effective training data, respectively. For a fair comparison, all parameters in active learning are the same as Q-AL-GPR in Table 1.

Compared with the AL-GPR and Q-AL-GPR methods, which use data from the whole space of positioning to construct the initial GPR model, the two new cases have worse performance due to the smaller coverage of the initial effective training dataset. This can be attributed to the heterogeneous illuminance pattern of each light source and the non-symmetric arrangement of light sources. In such a heterogeneous environment, an initial GPR model that covers a larger area has better generality which leads to better positioning accuracy. An interesting observation is that the performance of the “corner”, AL-GPR, and Q-AL-GPR cases are improved when more initial data are employed while the performance of the “center” case becomes worse. This can be explained by the fact that the central area has a higher SNR than the corner area. Therefore, the initial GPR model built on the low SNR corner samples is less accurate than the one built on the center samples. For the three cases that involve the low SNR samples from the corner area (i.e., AL-GPR, Q-AL-GPR, and “corner”), more initial data helps to mitigate the impact of noise. For the “center” case with high SNR samples, overfitting becomes the bottleneck due to the limited coverage of the initial data. As the Q-AL-GPR offers the best performance among all methods which share the same computational complexity, we will focus on the Q-AL-GPR in the following section to gain insight into the data-efficient training.

**Figure 3:**
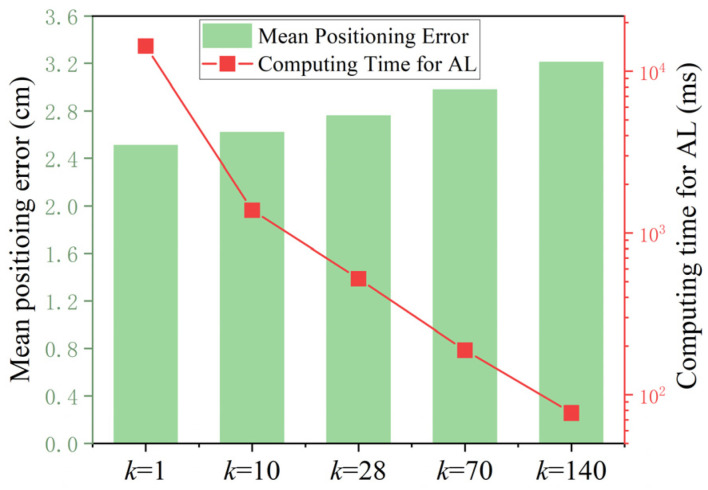
Mean positioning error and computing time for AL under different dataset update strategies (i.e., different *k* values).

As stated in section II, there are three adjustable parameters in the active learning for data-efficient training, including the number of initial (*m*) and final (E) effective training data and the number of new data (*k*) in each update of $D_{e}$. To properly set these parameters for optimized performance, extensive investigation has been carried out. We first test the system’s performance under different values of *k*. Figure 3 compares the mean positioning error and computation time for training under different dataset update strategies (i.e., *k* = 1/10/28/70/140 which corresponds to 140/14/5/2/1 iterations in the update of $D_{e}$) after 1000 runs. The other settings remain the same as those for Figure 2.

In general, when the dataset $D_{e}$ is updated iteratively with a smaller step (i.e., a smaller *k*), the positioning error gradually reduces whereas the computational time for AL increases significantly. The positioning accuracy is improved by 0.23 cm (0.45/0.59/0.7 cm) in terms of the mean positioning error when the number of iterations increases from 1 to 2 (5/14/140) for a fixed number of initial/final effective training data. This is attributed to the fact that more iterations allow for a finer process that selects the data with lower similarity. Nevertheless, the computation time increases for more iterations as the calculation of (4) is required in each iteration. The major complexity is attributed to the matrix inversion which has to be computed in each iteration due to the random nature of $D_{e}$. The measured computing time for active learning are 77.1/188.9/522.1/1382.2/14,314.9 ms for the five cases with 140/14/5/2/1 iterations, respectively, which is consistent with the complexity analysis in Section 2. To balance the computation complexity and positioning accuracy, five iterations are employed in the remaining test.

**Figure 4:**
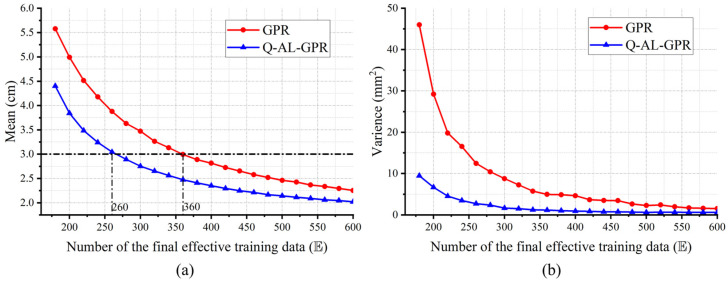
Empirical (**a**) mean and (**b**) variance of the average positioning error $\langle \varepsilon \rangle$ of each run under different sizes (E) of the finalized effective training dataset $D_{e}$.

Next, the impact of the number of final effective training data (E) on the performance of GPR-VLP is evaluated. Figure 4a,b show the mean and variance of the empirical distribution of the average positioning error $\langle \varepsilon \rangle$ of each run, respectively, when E increases from 180 to 800 in a step of 20. The number of initial effective training data is fixed at 160. The performance of the conventional GPR based on a randomly drawn training dataset of size E is also shown for compassion. The performance of both methods improves when a larger training dataset is used. Thanks to the active learning process, the Q-AL-GPR outperforms the GPR regardless of the value of E, which is more pronounced when a small/moderate number of effective training data is used. As the number of effective data used for training increases, the performance gap between the two methods is narrowed. For example, the mean (variance) of the empirical distribution is reduced by about 1 cm (15.3 mm^2^) for E = 220 when one uses the Q-AL-GPR instead of the GPR, whereas the above gain is 0.3 cm (1.3 mm^2^) for E = 550. This is because both methods draw training data from the same fixed set of candidates. The intersection of the training datasets for the two training methods becomes larger when E gradually approaches its limit (e.g., *N* = 1120 in our test). The same trend is observed from the perspective of variance of the empirical distribution. The positioning performance superiority of Q-AL-GPR over GPR leads to a significant enhancement of data efficiency in training at a certain target level of positioning accuracy. For example, the numbers of final effective training data are about 260 and 360 for Q-AL-GPR and GPR to achieve a mean error of 3 cm, respectively, which corresponds to an improvement of ~27.8% in data efficiency by active learning.

**Figure 5:**
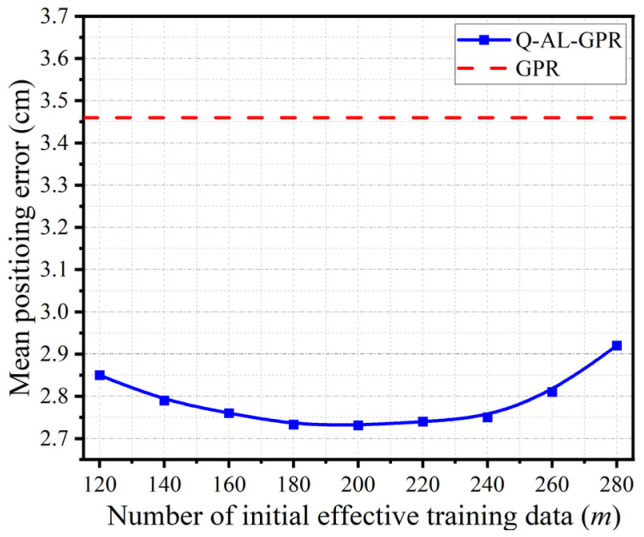
Mean positioning accuracy of Q-AL-GPR with different numbers of initial effective training data (*m*). The result of GPR with the same number of effective training data (E = 300) is shown by the dotted line for comparison.

The last parameter in active learning to be investigated is the number of initial effective training data (*m*). Figure 5 shows the mean positioning accuracy of Q-AL-GPR versus different values of *m* after 1000 runs. The number of final effective training data in active learning is set to 300. The performance of GPR with the same number of effective training data is shown by the dotted line for comparison.

The parabolic shape of the curve for Q-AL-GPR clearly shows that there exists an optimized value of *m* to achieve the highest mean positioning accuracy. When *m* is too small, the initialized GPR model with only a few randomly drawn data has a very poor generality and cannot reliably evaluate the similarity of data in $D_{*}$, which in turn disturbs the subsequent process of progressively finding the most valuable data for training in the active learning process. On the other hand, though the generality of the initialized GPR model is enhanced when *m* becomes larger, the number of additional training data that can be used to update the initialized GPR model is reduced as *m* approaches its limit E (e.g., 300 for Figure 5), which in turn negatively affects the performance of active learning. Therefore, the optimized value of *m* is determined by the interplay of the above two factors. As shown in Figure 5, the best performance of Q-AL-GPR is obtained when *m* is set to 200, which corresponds to an improvement of 33% in mean positioning accuracy over GPR (i.e., 2.73 cm versus 3.46 cm).

**Figure 6:**
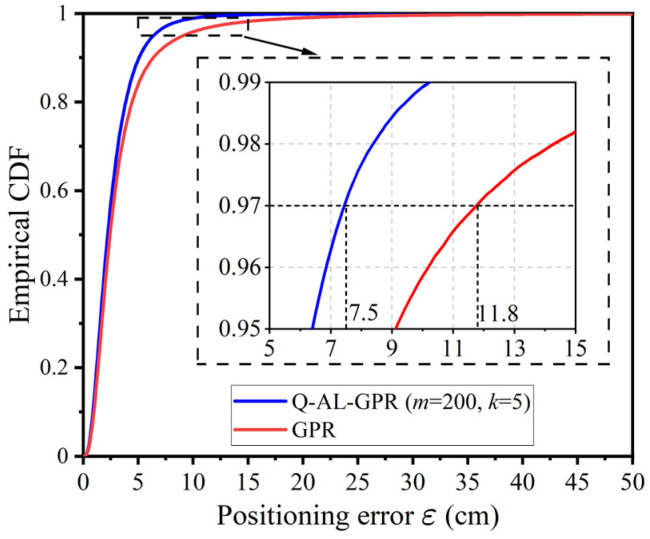
Empirical cumulative distribution function (CDF) of positioning error ε for Q-AL-GPR and CPR under 300 effective training data after 1000 runs.

To give a more comprehensive comparison of Q-AL-GPR and GPR, Figure 6 shows the empirical cumulative distribution function (CDF) of positioning error ε of the two methods with 300 effective training data after 1000 runs. The other parameters for Q-AL-GPR are *m* = 200 and *k* = 20. As shown in Figure 6, the curve of Q-AL-GPR is on the left side of that of GPR, which clearly shows the superiority of the active learning-based data-efficient training method over the conventional training method. For example, the positioning error of 97% of all tests is below 7.5 cm with Q-AL-GPR, while the value is 11.8 cm for GPR, which corresponds to an improvement of 36.4% in positioning accuracy.

**Figure 7:**
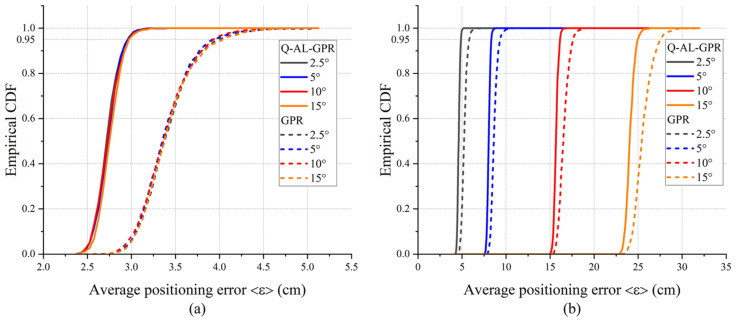
The empirical CDF of the average positioning error $\langle \varepsilon \rangle$ for GPR and Q-AL-GPR when the training data are collected (**a**) with or (**b**) without tilt. The test data are collected with a certain angle of receiver tilt in both scenarios.

Receiver tilting is an important factor in practice that affects the performance of the RSS-based VLP system. In the context of VLP with GPR, we have investigated two scenarios concerning the impact of receiver tilting: (A) the receiver is tilted when collecting both the training data and test data, and (B) the receiver is tilted when collecting test data while it is not tilted when collecting the training data. The receiver is tilted in the X-Z plane where the angle between the normal vector of the photodiode and the X-axis is varied from 0 to 15 degrees. The mean values of the average positioning error of 480 random locations after 1000 runs under different angles of tilt are shown in Table 2. The parameters for the positioning algorithms remain the same as in Figure 2 (*m* = 160, *k* = 28, and E = 300). To provide more information about the statistics of error, Figure 7 shows the empirical CDF of the average positioning error $\langle \varepsilon \rangle$ for the tests in Table 2.

As shown in Table 2, for both the GPR and Q-AL-GPR, the impact of receiver tilt is small in scenario A while it is much more obvious in scenario B, because the trained model is mismatched with the test environment in the latter one. Nevertheless, regardless of the angle of receiver tilt, the Q-AL-GPR always outperforms GPR in both scenarios. This shows that the effectiveness of the proposed method still holds when the receiver is tilted. The CDF curves in Figure 7 further show that the advantage Q-AL-GPR over GPR is more obvious when the tilt angle becomes larger.

**Figure 8:**
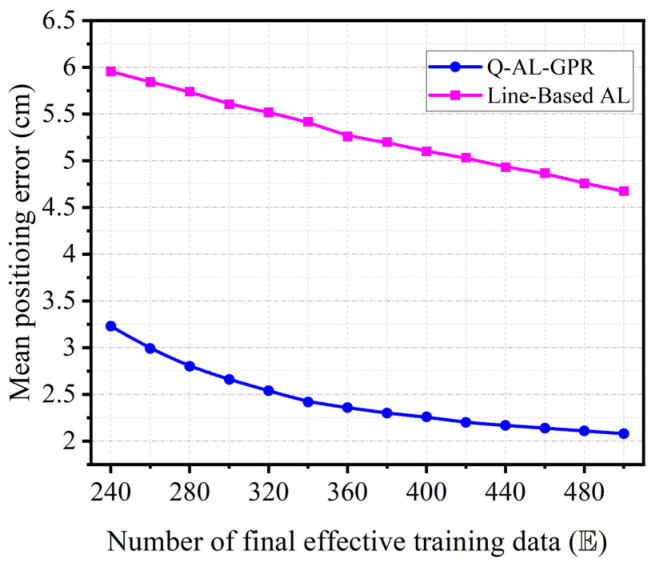
Mean positioning error versus different numbers of final effective training data (E) for the two training methods based on AL (i.e., Q-AL-GPR and line-based AL).

Finally, we compare the performance of the Q-AL-GPR with a line-based training method which also uses active learning. Figure 8 shows the mean positioning error of the above two training methods versus different numbers of final effective training data (E) after 1000 runs. For the line-based AL method, interpolation is used to sample data as in the previous study [16], and the sampling step along the line is set to 10 cm. The setting of Q-AL-GPR is the same as that in Figure 4. Our data-efficient method outperforms the line-based AL significantly in the whole range of tests (i.e., 240≤E≤500). Moreover, when one compares the red curve in Figure 4 (i.e., GPR without AL) and the pink curve in Figure 8, the data efficiency of the line-based AL method is even worse than the conventional training method with random sampling. The reason is that the line-based AL method is designed to improve the efficiency in collecting the training data (i.e., find a physical path as short as possible to collect all training data to achieve a certain level of positioning accuracy) rather than the data efficiency (i.e., find a training dataset as small as possible to achieve a certain level of positioning accuracy). From the perspective of data efficiency, the line-based AL method has poor performance as a large amount of data along the AL predicted lines is collected and added to the training dataset regardless of their similarity to the existing training data.

## 4. Conclusions

A data-efficient training method (namely Q-AL-GPR) for GPR-VLP systems is proposed and experimentally demonstrated. The proposed method uses the active learning methodology which gradually updates the effective training dataset with data of a low level of similarity. The experimental results of a three-dimensional VLP system verify that the performance of GPR-VLP can be improved by the proposed method when compared with the conventional training method based on a randomly drawn training dataset of the same size. The Q-AL-GPR also outperforms the AL-GPR with the same computational complexity thanks to the improved initialization process with a more uniform distribution of initialized training data. The impact of the parameters of active learning on the performance of Q-AL-GPR VLP is investigated, which reveals that (1) although the performance gain can be obtained by active learning regardless of the number of final effective training data, it is more pronounced for a small/moderate effective training dataset, (2) a moderate step size should be chosen for updating the effective training dataset to balance the conflicting requirements from the perspectives of positioning performance and computational complexity, and (3) there exists an optimized value for the number of initial effective training data due to the interplay of the reliability of the initialized GPR model and the flexibility in reshaping such a model via active learning. In terms of data efficiency in training, the required number of training data can be reduced by ~27.8% by Q-AL-GPR for a mean positioning accuracy of 3 cm when compared with GPR. The CDF analysis shows that the positioning error corresponding to the 97th percentile can be reduced from 11.8 cm (GPR) to 7.5 cm (Q-AL-GPR) with 300 effective training data, which corresponds to a ~36.4% improvement in positioning accuracy. The results show that the effectiveness of active learning still holds when the receiver is tilted. Our study also indicates that, under the same size of the training dataset, Q-AL-GPR is superior to the previous line-based AL training method which adapts the active learning to improve the data collection efficiency rather than data-efficiency in training.

## Figures and Tables

**Table 1:** The empirical mean/variance of $\langle \varepsilon \rangle$ for AL-GPR, Q-AL-GPR, and cases of “center” and “corner”.

**# of Initial Data (*m*)**		**120**	**140**	**160**	**180**	**200**
Mean (cm)	AL-GPR	2.88	2.84	2.80	2.77	2.76
	Q-AL-GPR	**2.85**	**2.79**	**2.76**	**2.74**	**2.73**
	center	3.29	3.38	3.51	3.70	3.96
	corner	3.51	3.46	3.43	3.45	3.45
Variance (mm^2^)	AL-GPR	2.32	2.08	2.04	1.90	1.76
	Q-AL-GPR	**2.17**	**1.87**	**1.83**	**1.75**	**1.67**
	center	4.18	4.45	4.98	6.16	8.42
	corner	6.07	5.48	5.71	5.61	5.07

**Table 2:** The empirical mean/variance of $\langle \varepsilon \rangle$ for GPR and Q-AL-GPR under different angles of receiver tilt using 300 effective training data.

* Scenario A	**Tilt Angle (degree)**	**0**	**2.5**	**5**	**10**	**15**
Mean (cm)	GPR	*3.46*	3.44	3.42	3.42	3.44
	Q-AL-GPR	*2.76*	2.75	2.76	2.76	2.78
Variance (mm^2^)	GPR	*8.77*	9.46	9.40	9.42	10.21
	Q-AL-GPR	*1.83*	1.67	1.73	1.93	1.72
* Scenario B	**Tilt angle (degree)**	**0**	**2.5**	**5**	**10**	**15**
Mean (cm)	GPR	*3.46*	5.28	8.68	16.59	25.59
	Q-AL-GPR	*2.76*	4.65	8.06	15.70	24.11
Variance (mm^2^)	GPR	*8.77*	10.12	14.32	35.60	115.21
	Q-AL-GPR	*1.83*	2.21	3.29	6.87	25.81

* Scenarios A (B) refers to the case when the training data are collected with (without) tilt. The receiver is tilted by a certain angle when collecting the test data in both scenarios.

## Data Availability

Data underlying the results presented in this paper are not publicly available at this time but may be obtained from the authors upon reasonable request.
